# Unveiling the crucial roles of abscisic acid in plant physiology: implications for enhancing stress tolerance and productivity

**DOI:** 10.3389/fpls.2024.1437184

**Published:** 2024-11-21

**Authors:** Weiliang Mo, Xunan Zheng, Qingchi Shi, Xuelai Zhao, Xiaoyu Chen, Zhenming Yang, Zecheng Zuo

**Affiliations:** ^1^ Jilin Province Engineering Laboratory of Plant Genetic Improvement, College of Plant Science, Jilin University, Changchun, China; ^2^ College of Animal Science and Technology, Guangxi Key Laboratory of Animal Breeding, Disease Control and Prevention, Guangxi University, Nanning, Guangxi, China

**Keywords:** abscisic acid, molecular mechanisms, biosynthesis, catabolism, signaling

## Abstract

Abscisic acid (ABA), one of the six major plant hormones, plays an essential and irreplaceable role in numerous physiological and biochemical processes during normal plant growth and in response to abiotic stresses. It is a key factor in balancing endogenous hormones and regulating growth metabolism in plants. The level of ABA is intricately regulated through complex mechanisms involving biosynthesis, catabolism, and transport. The functionality of ABA is mediated through a series of signal transduction pathways, primarily involving core components such as the ABA receptors PYR/PYL/RCAR, PP2C, and SnRK2. Over the past 50 years since its discovery, most of the genes involved in ABA biosynthesis, catabolism, and transport have been characterized, and the network of signaling pathways has gradually become clearer. Extensive research indicates that externally increasing ABA levels and activating the ABA signaling pathway through molecular biology techniques significantly enhance plant tolerance to abiotic stresses and improve plant productivity under adverse environmental conditions. Therefore, elucidating the roles of ABA in various physiological processes of plants and deciphering the signaling regulatory network of ABA can provide a theoretical basis and guidance for addressing key issues such as improving crop quality, yield, and stress resistance.

## 1 Introduction

Abscisic acid (ABA), a sesquiterpenoid compound containing 15 carbon atoms, is now recognized as one of the six classical plant hormones that regulate plant growth and physiology. In the 1960s, Torsten Hemberg discovered a plant growth inhibitory substance that was soluble in both water and ether, which was crucial for maintaining dormancy in the tubers of potatoes *Solanum tuberosum* (Hemberg, 1949a) and the terminal buds of *Fraxinus* (Hemberg, 1949b). This was the first time that ABA was identified in plants. In 1963, Philip Wareing isolated the growth-inhibiting substance, which he named “dormin” due to its significant role in bud dormancy, from the buds of *Acer platanoides* (Eagles and Wareing, 1963). In the same year, Frederick Addicott applied this substance in amounts as low as 0.01 μg per abscission zone, finding that it accelerated abscission, so he discovered a substance that controlled the abscission of cotton (*Gossypium hirsutum*) young fruits and thus named it abscisin II (Ohkuma et al., 1963). Subsequently, Addicott found that abscisin II also could promote leaf abscission in cotton seedlings and inhibit the elongation of oat (*Avena sativa*) coleoptiles induced by indoleacetic acid (Ohkuma et al., 1963). Later, it was discovered that dormin and abscisin II were the same chemical compound, which was then named abscisic acid (Chen K. et al., 2020). Despite the term “abscisic” in the name of abscisic acid, as research advanced, it has been found that ABA does not directly regulate the formation of abscission layers in senescing plant tissues but rather exerts an indirect influence by elevating ethylene (ETH) levels. On the contrary, it regulates the physiological processes within the plant before abscission (Cracker and Abeles, 1969; Ahmad and Guo, 2019; Altaf et al., 2024).

**Figure 1:**
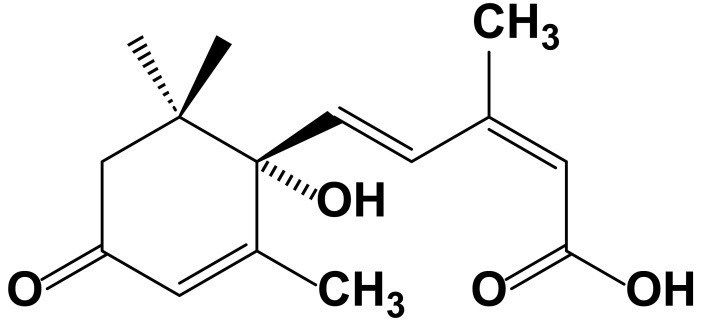
Structure of abscisic acid (ABA). ABA has a chemical formula of C_15_H_20_O_4_ and features a molecular structure comprising an acrylic acid group and an isopentenyl alcohol group. Additionally, it contains a carboxyl group (–COOH) and a hydroxyl group (–OH). The biologically active form primarily exists as dextrorotatory (+)-*S*-ABA. Overall, the structure of abscisic acid is relatively straightforward, yet it plays a crucial regulatory role in plant growth and adaptation to the environment.

**Figure 2:**
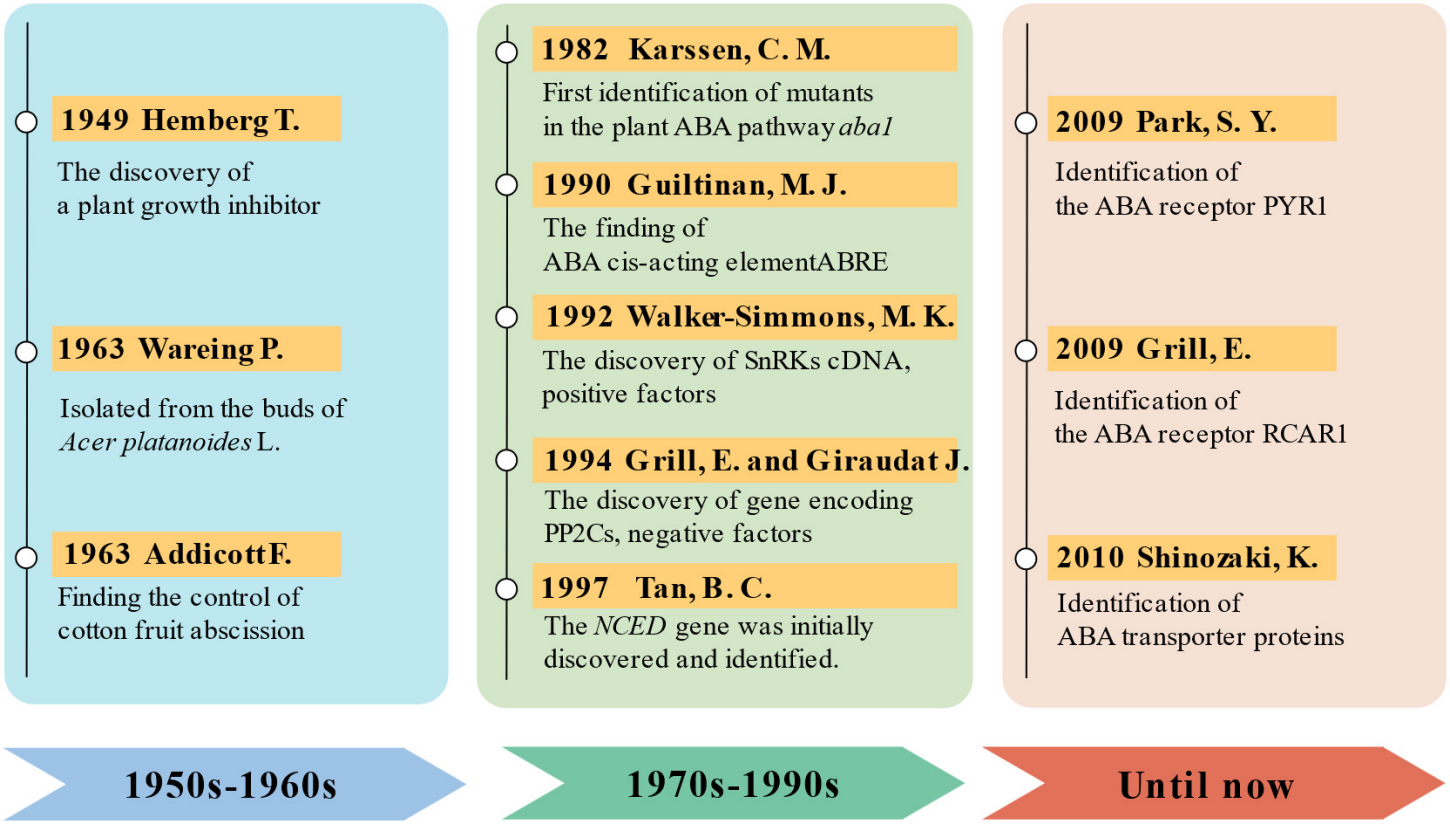
The history of abscisic acid (ABA) research development. 1950s to 1960s: discovery and early research on ABA. Hemberg discovered a plant growth inhibitor that was soluble in water and ether, initiating preliminary studies on physiological effects of ABA (Hemberg, 1949a, 1949b). It was found that ABA plays a crucial regulatory role in processes such as seed dormancy and germination, root growth, water regulation, flowering, and fruit ripening (Eagles and Wareing, 1963; Ohkuma et al., 1963). 1970s to 1990s: research on aba biosynthesis and metabolism. Following the discovery of *Arabidopsis* mutants defective in ABA biosynthesis pathways (Koornneef et al., 1982), a combination of molecular biology, genetic engineering, and forward and reverse genetics gradually revealed the pathways involved in ABA biosynthesis (Guiltinan et al., 1990; Anderberg and Walker-Simmons, 1992; Leung et al., 1994; Meyer et al., 1994; Tan et al., 1997). Until now: advancements in molecular biology and beyond. Since the 2000s, significant progress has been made in the study of ABA biosynthesis, metabolism, receptors, and signaling pathways, owing to advancements in molecular biology and bioinformatics technologies. Particularly, the identification of ABA receptors has linked plant perception of ABA to core signaling components for the first time (Ma et al., 2009; Park et al., 2009; Kuromori et al., 2010). Through techniques such as genetic engineering, genomics, and proteomics, the complexity and diversity of ABA signaling pathways have been unveiled. Researchers have also explored the regulatory mechanisms of ABA in plant growth, development, stress responses, and stress tolerance, providing crucial theoretical foundations for plant biology and agricultural production.

Natural ABA exists as enantiomers, with the biologically active form predominantly being the dextrorotatory (+)-*S*-ABA (**Figure 1**) (Zaharia et al., 2005; Dörffling, 2015). In the 1980s, ABA pathway mutants of plants were first identified, with the *aba1*-deficient mutant of *Arabidopsis thaliana* being among the earliest. This mutant led to the identification of the first enzyme in the abscisic acid biosynthetic pathway, zeaxanthin epoxidase (ZEP) (Koornneef et al., 1982). This discovery provided crucial insights for further research into the biosynthesis of abscisic acid. Since then, nearly all genes encoding the core components of ABA biosynthesis and signal transduction have been identified through the isolation of deficient mutants (Schwartz et al., 2003; Taylor et al., 2005). Subsequently, comprehensive exploration of ABA entered a period of rapid development, leading to significant advances in identifying the mechanisms and genes involved in ABA metabolism, transport, and signal transduction (Riera et al., 2005; Kumar et al., 2022). Through molecular biology, biochemistry, genetics, and other methods, numerous important enzymes, transcription factors, and the genes encoding them have been discovered. Examples include ABA insensitive (ABI) 1 and ABI2, which are members of group A of Protein Phosphatases 2C (PP2C) (Merlot et al., 2001; Sun et al., 2011; Ludwików, 2015) and core transcription factors such as ABI3 (a B3 domain transcription factor) (Delmas et al., 2013), ABI4 [an APETALA2-type (AP2) transcription factor] (Huang et al., 2017), and ABI5 [a basic leucine zipper (bZIP) transcription factor] (Nie et al., 2022). In plants, ABA exerts its functions through recognition by intracellular receptors. However, it was not until 2009 that ABA receptors were independently discovered by research teams from the United States and Germany, utilizing entirely different research methods, and published in the same issue of the journal *Science*. The Cutler research group, from the perspective of chemical genetics, conducted genetic screening using a selective ABA antagonist (pyrabactin), ultimately identifying a cyclase subfamily member belonging to the larger superfamily containing Steroidogenic Acute Regulatory-related Lipid Transfer (START) domains, termed Pyrabactin Resistance 1 (PYR1) (Park et al., 2009). The Grill research group from Germany utilized the yeast two-hybrid system in genetic approaches, using the Mg^2+^- and Mn^2+^-dependent PP2C members ABI1 and ABI2 as bait to screen for proteins interacting with them and identifying a protein, naming it Regulatory Component of ABA Receptor 1/3 (RACR1/3) (Ma et al., 2009). The discovery of ABA receptors is one of the most significant advancements in the field of plant biology in recent years. It has linked plant perception of ABA with core signaling components, leading to extensive molecular biology, biochemistry, genetics, and physiology research, elucidating the functions of ABA receptors and the role of the ABA signaling pathway in plant growth, development, and stress responses (Rodriguez et al., 2019; Fidler et al., 2022). Since the discovery of ABA in the last century and the subsequent identification of its receptors, research on ABA has reached unprecedented heights (**Figure 2**).

ABA is involved in regulating various stages of plant growth and development, including seed maturation, dormancy, germination, seedling growth, stomatal movement, and the transition from vegetative to reproductive growth (Yan and Chen, 2017; Gupta et al., 2022; Kavi Kishor et al., 2022). Additionally, ABA plays a significant role in the plant response to various environmental stresses, for example, drought, salinity, low temperature, osmotic stress, mechanical damage, and biotic stresses such as diseases and pests (Matsui et al., 2008; Lievens et al., 2017; Vishwakarma et al., 2017; Muhammad Aslam et al., 2022; Waadt et al., 2022). The realization of multiple physiological functions of ABA relies on the dynamic equilibrium of intracellular ABA levels and its intricate regulatory mechanisms, consisting of enzymes involved in ABA synthesis and metabolism, cellular recognition of ABA, and subsequent activation of downstream transcription factors (Chen K. et al., 2020; Sun et al., 2020). Since the discovery of the first mutant in the ABA biosynthetic pathway in the 1980s, over 200 loci involved in ABA metabolism, transport, and signaling have been identified through the integration of molecular biology, genetic engineering, and forward and reverse genetic approaches (Schwartz et al., 2003; Lim et al., 2022). The intracellular concentration of ABA in plants is fundamental for initiating ABA-mediated signaling during plant development and in response to environmental changes. The levels of ABA fluctuate continuously with physiological and environmental conditions, and these concentration changes determine the functionality of ABA in plant physiology and development (Merilo et al., 2015; Abhilasha and Roy Choudhury, 2021).

ABA plays a crucial role in plant growth and agricultural production. With the growing global population and increasing environmental challenges, enhancing plant resilience has become essential to meet the growing global food demands. Therefore, in-depth research into the dynamic equilibrium and molecular mechanisms of ABA is of paramount importance, which aims to utilize genetic engineering techniques to bolster plants’ stress resistance, foster agricultural abundance, and ameliorate human production and livelihoods. However, our current understanding of the complex regulatory networks remains limited. Therefore, our review provides a summary of classic models of ABA biosynthesis, metabolism, catabolism, signaling transduction, and transport, while incorporating the latest research advancements, to offer new insights into the intricate regulatory network of ABA, with a particular focus on potential molecular mechanisms.

## 2 The biosynthesis of ABA

Early studies have suggested that roots were the primary ABA synthetic organs in plants (Tardieu and Davies, 1993), but subsequent research has indicated that leaves are also major sites for ABA synthesis (Zhang et al., 2018), intracellular ABA biosynthesis mainly occurs in organelles such as plastids, the endoplasmic reticulum, and vacuoles (Finkelstein, 2013). Its synthesis chiefly occurs through two pathways: the carotenoid indirect synthesis pathway and the terpenoid direct pathway.

The carotenoid indirect pathway is the predominant route for ABA biosynthesis in higher plants, and extensive research and summarization have been conducted on the intermediates and enzymes involved. In simple terms, the biosynthesis pathway in plants begins with the methylerythritol 4-phosphate (MEP) pathway, in which key intermediates are synthesized in plastids such as β-carotenoid. Subsequently, through a series of isomerization and cleavage reactions, xanthoxin is formed, catalyzed by the central rate-limiting enzyme 9-*cis*-cyclocarotenoid dioxygenase (NCED). Xanthoxin is then further oxidized in the cytoplasm to yield ABA ultimately (Cowan, 2001; Nambara and Marion-Poll, 2005; Finkelstein, 2013; Cardoso et al., 2020; Lim et al., 2022; Wu W. et al., 2023).

**Figure 3:**
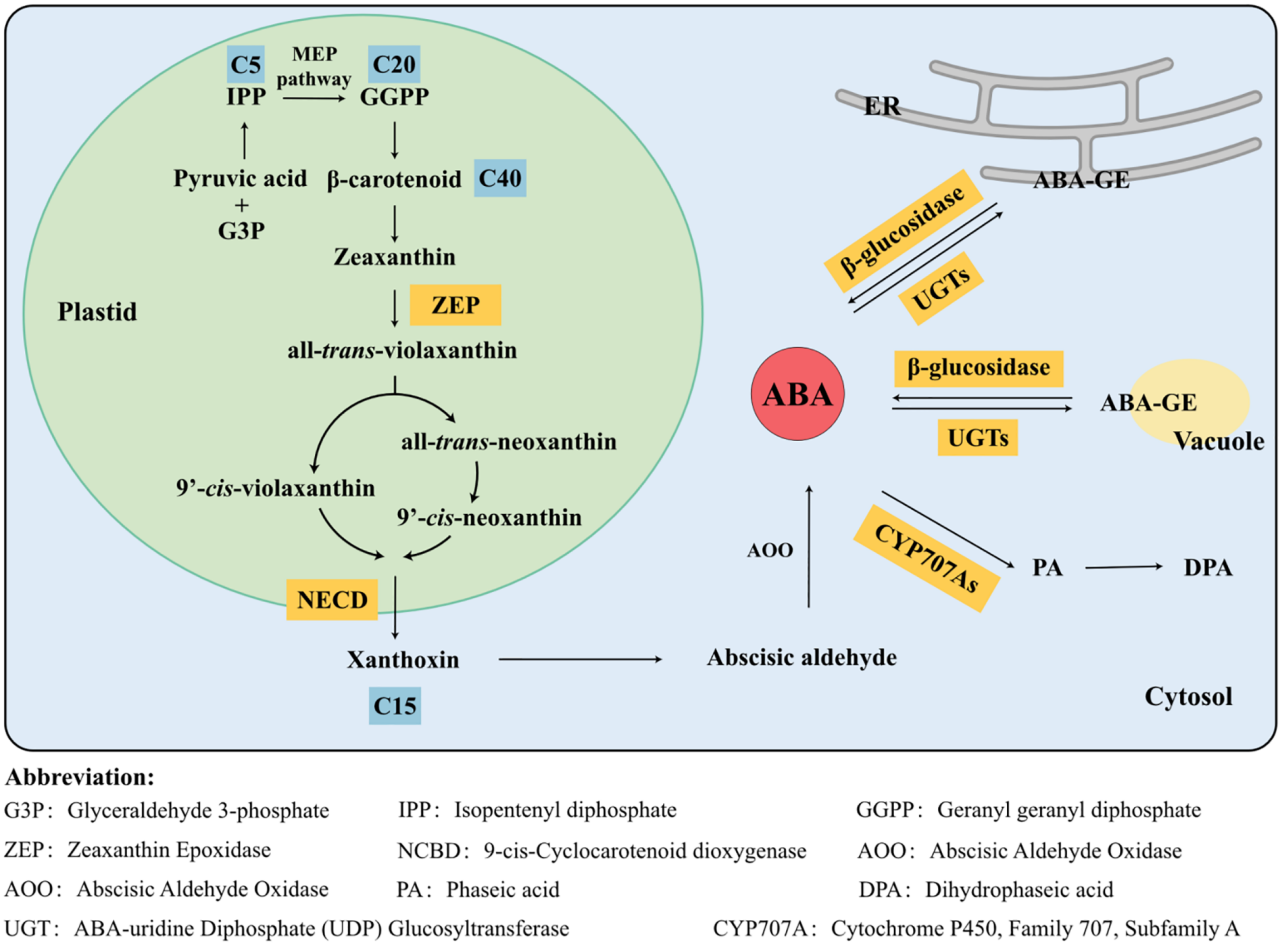
Biosynthesis and catabolism metabolic pathways of abscisic acid (ABA) in higher plants. The biosynthesis and catabolism pathways of ABA represent pivotal aspects of plant physiology research. ABA biosynthesis predominantly occurs in plastids and cytosol, with C40 β-carotenoids serving as the primary precursors. 9-*cis*-Cyclocarotenoid dioxygenase (NCED) acts as a crucial rate-limiting enzyme in the synthesis pathway. Additionally, hydrolysis of ABA-GE provides free ABA, constituting another essential mechanism for regulating ABA concentration in plants. ABA catabolism primarily involves two pathways: hydroxylation and glucosylation. With the assistance of enzymes, ABA is metabolized into inactive metabolites, which no longer exhibit biological activity in plant growth and development.

The specific synthesis pathway is as follows (**Figure 3**): in plastids, pyruvic acid and glyceraldehyde 3-phosphate (G3P) are converted through the MEP pathway into key intermediates like isopentenyl diphosphate (IPP) and geranylgeranyl diphosphate (GGPP), leading to the formation of the C40 intermediate β-carotenoid. GGPP serves as a common C20 precursor for both gibberellin (GA) and ABA (Salazar-Cerezo et al., 2018). Subsequently, β-carotenoid undergoes consecutive oxidation and hydroxylation, catalyzed by enzymes such as β-carotene hydroxylase to form zeaxanthin. Zeaxanthin is converted to all-*trans*-violaxanthin by ZEP. It can either be isomerized to 9′-*cis*-violaxanthin or first converted to all-*trans*-neoxanthin by neoxanthin synthase and then isomerized to 9′-*cis*-neoxanthin. NCED catalyzes the cleavage of the C_11_ and C_12_ bonds of 9′-*cis*-violaxanthin and 9′-*cis*-neoxanthin, leading to the formation of xanthoxin, which is considered the rate-limiting and crucial step in ABA biosynthesis (Taylor et al., 2000). In the cytoplasm, xanthoxin is first oxidized to abscisic aldehyde, which is then further oxidized to form ABA. These two sequential reactions are catalyzed by a short-chain alcohol dehydrogenase (SDR) and abscisic aldehyde oxidase (AAO).

The key reactions for ABA biosynthesis occur in the steps following β-carotenoid formation in higher plants. One of the regulatory enzymes widely recognized as playing a critical role in ABA biosynthesis in higher plants is NCED (Tan et al., 2003; Changan et al., 2023; Chen et al., 2024). The *NCED* gene was first discovered and identified in the *viviparous* seed mutants of maize. Tan et al. identified two alleles of a new mutant, *viviparous 14* (*vp14*), through transposon mutagenesis. They found that the ABA content in the embryos of this mutant was 70% lower during development compared to the wild type, indicating a severe defect in ABA biosynthesis (Tan et al., 1997). Different *NCED* genes have now been cloned from various species, all of which belong to the *Carotenoid Cleavage Dioxygenase* (*CCD*) family, and the related mutants exhibit phenotypes associated with ABA deficiency (Ohmiya, 2009). In *Arabidopsis*, the *CCD* family consists of nine members, but only five genes are engaged in ABA synthesis: *NCED2*, *NCED3*, *NCED5*, *NCED6*, and *NCED9* (Neill et al., 1998; Tan et al., 2003; Ohmiya, 2009). The contributions of *NCED*s to ABA synthesis during plant growth and development vary. Gene expression analysis indicated that *AtNCED2* and *AtNCED3* had higher expression levels in roots and leaves but remained low during seed development. In contrast, the expression levels of *AtNCED5*, *AtNCED6*, and *AtNCED9* increased during the later stages of seed maturation (Tan et al., 2003; Ma et al., 2018). Based on site-specific and functional divergence analyses, Siva et al. found that *NCED* genes promoted tissue-specific functional diversification within the *NCED* subfamily, which enabled plants to withstand various environmental stress conditions, ensuring their survival. Additionally, they discovered that the evolution of *NCED* genes occurred through events of duplication, diversification, and exon–intron loss (Priya and Siva, 2015).

*NCED* genes have been cloned and studied in various plants. On the one hand, an increasing body of evidence suggests that its role in enhancing plant stress resistance, including against heavy metal stress, should not be underestimated. On the other hand, in plant growth and development, seed dormancy, and germination, the regulation of ABA content by NCED constitutes a significant physiological activity. **Table 1** summarizes the functions of *NCED* genes over recent years. Apart from the aforementioned points, NCED demonstrates efficacy across numerous other domains. The role of *NCED* in regulating plant height was first documented in transgenic sweet potatoes (*Ipomoea batatas*), where the Huo team found that *IbNCED1* regulates plant height and development by controlling the ABA and GA signaling pathways (Zhou et al., 2023). The overexpression of *OsNCED3* in rice *Oryza sativa* demonstrated potential value in rice breeding by increasing its resistance against the brown planthopper (*Nilaparvata lugens*) (Sun et al., 2022). In wheat (*Triticum aestivum*), *Myb10-D* slowed down germination by activating *NCED* to enhance the early biosynthesis of ABA during seed development (Lang et al., 2021). Recent studies have extensively explored *NCED* cloning and function in both agricultural and economic crops, including soybean (*Glycine max*) (Molinari et al., 2020), maize (*Zea mays*) (Li et al., 2019), wheat (Tong et al., 2017), and pear (*Pyrus sinkiangensis*) (Liu et al., 2023). The studies above have collectively underscored the pivotal role of NCED as a key rate-limiting enzyme in the ABA biosynthesis pathway. By regulating ABA accumulation and content, NCED profoundly influences various aspects of plant physiology including stress resilience, seed dormancy, and fruit ripening.

**Table 1:** The functions of NCED in different species.

Function	Species	NCED	Specific role	Reference
Stress resistance	Rice	OsNCED1	Positive regulation of heat tolerance in rice seedlings.	(Zhang et al., 2022)
		OsNCED3	Improvement of drought tolerance.	(Huang et al., 2018; Changan et al., 2023)
		OsNCED5	Enhancement of salt and water stress tolerance.	(Huang et al., 2019)
	*Prunus mume*	PmNCED3 PmNCED8 PmNCED9	Indispensable in the response to cold stress.	(Chen et al., 2023)
Resisting heavy metals	Circus (*Citrus limonia*)	ClNCED1 ClNCED3 ClNCED5	Closure of leaf stomata and an increase in ABA concentration under aluminum (Al) stress.	(Gavassi et al., 2021)
	Ramie (*Boehmeria nivea*)	BnNCED3 BnNCED5	Upregulation of *NCED* expression and increased ABA concentration under chromium (Cd) stress.	(Chen et al., 2021)
Plant growth and development	Tomato (*Solanum lycopersicum*)	SlNCED1	*SlNCED1* activity peaks during the fruit color change stage. In *SlNCBD1*-RNAi plants, the development of pistils and fruit set rate is impaired, with abnormal anther development and the production of a large quantity of abnormal pollen.	(Wang X.H. et al., 2021; Efremov et al., 2023)
	Raspberry (*Rubus idaeus*)	RiNCED1	Both ABA content and *RiNCED1* expression consistently increase during fruit ripening.	(Álvarez et al., 2023)
	Sweet cherry (*Prunus avium*)	PavNCED5	PavNCED5 plays a significant role in flower bud dormancy.	(Wang et al., 2023)
	Peach (*Prunus persica*)	PpNCED1 PpNCED5	The *PpNCED1* and *PpNCED5* genes control ABA biosynthesis during peach maturation and senescence by modulating ROS signals.	(Wang P.F. et al., 2021)
	Gastrodia (*Gastrodia elata*)	GeNCED2	Downregulating the expression of *GeNCED2* can reduce ABA synthesis and block germination-inhibiting signals, thereby breaking seed dormancy.	(Ren et al., 2021)
	Terrestrial orchids (*Phaius tankervilleae*)	PtNCED1	The involvement of *PtNCED1* in regulating endogenous ABA levels during seed and tuber development.	(Lee et al., 2018)

NCED, 9-*cis*-cyclocarotenoid dioxygenase; ABA, abscisic acid; ROS, reactive oxygen species.

ZEP, a crucial enzyme in ABA biosynthesis, is a bifunctional monooxygenase located on the stromal side of the thylakoid (Yamamoto et al., 1962; Agrawal et al., 2001; Barrero et al., 2005). ZEP is the initial enzyme discovered in the ABA biosynthesis pathway, catalyzing the conversion of C40 carotenoids to zeaxanthin epoxide. The first gene encoding *ZEP* was identified in the *aba1* mutant of *Arabidopsis*, which showed a shortened seed dormancy period, reduced ABA levels during development and maturation, and an inability to epoxidize zeaxanthin into violaxanthin (Koornneef et al., 1982). Subsequent research found that the *Nicotiana plumbaginifolia aba2*-deficient mutant exhibited a similar phenotype, whose characterization was an inability to carry out the first step of ABA synthesis. The tobacco *Nicotiana tabacum ABA2* gene could complement the functional defects of both the *Arabidopsis aba1* mutant and the *N. plumbaginifolia aba2* mutant, implying that *Arabidopsis aba1* and *N. plumbaginifolia aba2* were homologous genes. Additionally, the expression of the ABA2 protein *in vitro* demonstrated its ability to catalyze the formation of zeaxanthin epoxide from violaxanthin, further confirming the identification of the *ZEP* gene successfully (Marin et al., 1996).

The *ZEP* gene has been successively cloned and functionally characterized in plants such as rapeseed (*Brassica napus*) (Ye et al., 2024), pepper (*Capsicum annuum*) (Lee et al., 2021), cotton (*Gossypium arboreum*) (Wu and Zhou, 2018), and tomato (Galpaz et al., 2008). ZEP plays a pivotal role in the consecutive epoxidation of zeaxanthin, an essential step in ABA biosynthesis. *ZEP* mutants exhibit lower ABA levels during plant growth and development, including under stress conditions, with minimal ABA upregulation (Xiong et al., 2002; Mizokami et al., 2015). In contrast, numerous studies have shown that the overexpression of *ZEP* can enhance plant stress tolerance, indicating that *ZEP* is crucial in ABA-mediated stress response mechanisms (Park et al., 2008). Additionally, ZEP also plays an indispensable role in carotenoid pigmentation. Therefore, ZEP levels also influence the color of the fruit (Liu Y.J. et al., 2020). ZEP, together with violaxanthin de-epoxidase (VDE), participates in the xanthophyll cycle, contributing to light harvesting and protecting plants from photoinhibition (Demmig-Adams and Adams, 1996). In summary, ZEP is crucial for ABA and carotenoid biosynthesis. Some of the roles of ZEP are listed in **Table 2**.

**Table 2:** The roles of ZEP.

Species	Function		Reference
Rice	Enhancement of drought tolerance with an eightfold increase in ZEP expression under drought stress.		(Changan et al., 2023)
Wheat	Silencing of the *TaZEP* gene significantly reduced tolerance to nitrogen deficiency.		(Kang et al., 2019)
Pepper	Influence on fruit color	Compared to the wild-type variety, *ZEP* mutant fruits exhibited a bright red color.	(Lee et al., 2024)
		*ZEP* gene activity determines the orange or yellow coloration of mature pepper fruits.	(Lee et al., 2021)
Chinese cabbage (*Brassica rapa*)		The gene encoding *ZEP* may be identified as a candidate gene for the deep yellow flower trait.	(Yang et al., 2022)
Rice	Involved in the xanthophyll cycle, contributing to light harvesting and protecting plants from photoinhibition.		(Wu H. et al., 2023)

ZEP, zeaxanthin epoxidase.

Additionally, there is a physiologically inactive conjugated form called ABA-glucosyl ester (ABA-GE); ABA-GE is synthesized through the conjugation of ABA with glucose, and the specific synthesis pathway is detailed later in the section on ABA metabolism. The hydrolysis of ABA-GE into the active free form of ABA is also crucial, as this process is vital for the regulation of plant growth and development by ABA (Dong et al., 2015). This step is carried out by the ABA-specific β-glucosidase of plants, which hydrolyzes the inactive ABA-GE to form active ABA (Dietz et al., 2000). ABA-GE is considered a storage and transport form of ABA (Cutler and Krochko, 1999), accumulating in the vacuole or apoplast (Lehmann and Glund, 1986; Dietz et al., 2000). To date, four β-glucosidases in *Arabidopsis* (AtBG1, AtBG2, BGLU10, and BGLU18) have been found to catalyze the conversion of ABA-GE to ABA (Lee et al., 2006; Han et al., 2020). The maize genome contains 26 genes encoding β-glucosidases, and signal peptide analysis indicated that these enzymes were localized to the plastids, mitochondria, cytosol, and vacuoles (Gómez-Anduro et al., 2011). *Arabidopsis atbg1* mutants showed severe ABA deficiency, while AtBG1 overexpression led to ABA accumulation. In contrast, *atbg2* mutants exhibited milder phenotypes. These findings suggest distinct roles for the homologous β-glucosidases in different physiological responses (Lee et al., 2006). AtBG1 is situated within the endoplasmic reticulum (ER), featuring an ER retrieval motif at its C-terminus. Conversely, AtBG2 carries a vacuole-targeting motif at its N-terminus. These observations indicated that two homologous β-glucosidases produced ABA in different cellular compartments (ER and vacuole), thereby forming an active reservoir for swift physiological responses under stress conditions (Lee et al., 2006). Moreover, there was an acceleration in the aggregation of AtBG1 in *Arabidopsis* under drought stress, leading to increased enzymatic activity in hydrolyzing ABA-GE and subsequently elevating the ABA content. Simultaneously, AtBG1 activity displayed diurnal oscillations, aligning with the trend of higher ABA levels during the day and lower levels at night (Lee et al., 2006). Os3BGlu6 of rice has been shown to hydrolyze ABA-GE into ABA *in vitro*. Mutant plants lacking *Os3BGlu6* exhibited stunted growth, accompanied by decreased leaf ABA levels and photosynthesis rates, ultimately leading to reduced drought resistance in rice (Wang et al., 2020). These studies have highlighted the role of β-glucosidases in rapidly converting ABA-GE into active ABA, enabling plants to meet their stress-related demands.

Certainly, research on the biosynthetic pathway of ABA is ongoing, and a recent study has discovered an alternative ABA biosynthetic pathway that may operate independently of ZEP and could be conserved in higher plants. Jia et al. demonstrated through combined genetic and biochemical experiments that the pathway initiates upstream of zeaxanthin, with a series of carotenoid cleavage products (including apocarotenoids, β-apo-11-carotenal, 9-*cis*-β-apo-11-carotenal, 3-OH-β-apo-11-carotenal, and 9-*cis*-3-OH-β-apo-11-carotenal) serving as intermediates. Using labeled compounds, they found that in *Arabidopsis* and rice, β-apo-11-carotenal, 9-*cis*-β-apo-11-carotenal, and 3-OH-β-apo-11-carotenal were successively converted to 9-*cis*-3-OH-β-apo-11-carotenal, xanthoxin, and eventually to ABA. Treatment of *Arabidopsis* with β-apo-11-carotenoids revealed their biological functions resembling those of ABA, such as maintaining seed dormancy and inducing the expression of ABA-responsive genes. Transcriptome analysis showed a significant overlap between genes differentially expressed in response to β-apo-11-carotenoids and those induced by ABA but also suggested that these compounds exhibit ABA-independent regulatory activities. This discovery expands our understanding of ABA biosynthesis and provides new insights into the apocarotenoid metabolic network in plants (Jia K.P. et al., 2022).

Another direct biosynthetic pathway, also known as the terpenoid pathway or C15 direct pathway, begins with the condensation of mevalonic acid (MVA) and IPP to form the C15 precursor farnesyl pyrophosphate (FPP). Subsequently, FPP undergoes cyclization and oxidation reactions to directly form 15-carbon ABA (Hirai et al., 2000). It is currently believed that this pathway primarily exists in fungi (Crocoll et al., 1991), but the biosynthetic pathways of ABA vary across different species (Crocoll et al., 1991; Hirai et al., 2000; Yamamoto et al., 2000; Inomata et al., 2004; Takino et al., 2018, 2019). Fungi such as *Botrytis cinerea* (Takino et al., 2018, 2019), *Cercospora cruenta* (Yamamoto et al., 2000; Inomata et al., 2004), and *Rhizopus nigricans* (Crocoll et al., 1991) have been extensively studied in this regard.

Currently, in lower plants, including numerous eukaryotic algae and some species of cyanobacteria that produce ABA, the biosynthetic pathway remains incompletely understood. ABA biosynthesis has not been detected in cyanobacteria and some red algae, indicating the absence of the crucial precursor epoxycarotenoids violaxanthin and neoxanthin required for ABA biosynthesis (Takaichi et al., 2009; Hartung, 2010; Takaichi, 2011). Thus, there may be an alternative ABA biosynthetic pathway in lower plants that differs from that in higher plants. From an evolutionary perspective, this pathway may also be conserved in higher plants, being activated under certain stress conditions to produce ABA in response to adverse environmental changes.

## 3 The catabolism of ABA

The levels of ABA hormones are determined by the rates of both catabolism and biosynthesis, which are crucial for regulating plant growth and metabolism in response to different environmental conditions. Compared to the biosynthetic pathways, the catabolic pathways of ABA are simpler, primarily involving hydroxylation and glycosylation processes (Zeevaart and Creelman, 1988; Ng et al., 2014). The two pathways involve the hydroxylation of ABA mediated by Cytochrome P450-type monooxygenases and the glycosylation of ABA mediated by glucosyltransferases in *Arabidopsis* (Cutler and Krochko, 1999) (**Figure 3**).

In the hydroxylation pathway, ABA is hydroxylated at three positions on its ring structure (C-7′, C-8′, and C-9′), resulting in various metabolites. The 8′-hydroxylation is the primary hydroxylation route and a crucial step in the main catabolic pathway. It is catalyzed by enzymes Cytochrome P450, Family 707, Subfamily A (CYP707A), a key enzyme in ABA metabolism, from the Cytochrome P450 monooxygenase family (Ng et al., 2014). Hydroxylated ABA is unstable and subsequently undergoes spontaneous isomerization to phaseic acid (PA), which has reduced biological activity compared to ABA. PA is further processed at the 4′ position by phaseic acid reductase (PAR) to produce dihydrophaseic acid (DPA), which is biologically inactive. PA and DPA are the most abundant ABA catabolites in plants. There may be other similar metabolites, such as epi-DPA (Ng et al., 2014; Dong et al., 2015).

Compared to the well-documented 8′-hydroxylation pathway, the catabolic pathways involving ABA 7′-hydroxylation and 9′-hydroxylation are less well understood. While studies have investigated the metabolic mechanisms of these pathways, they are not as clearly elucidated as the 8′-hydroxylation pathway. 7′-Hydroxylation ABA was isolated from the culture medium of smooth bromegrass (*Bromus inermis*) cell suspension cultures (Hampson et al., 1992). Currently, there are very few studies on the 7′-hydroxylation pathway with limited knowledge that it may exist as an important secondary metabolite in various plants (Hampson et al., 1992; Nambara and Marion-Poll, 2005). In barley (*Hordeum vulgare*) half seeds, 7′-hydroxylation ABA exhibited inhibitory effects on both GA_3_ and α-amylase activity, albeit less potent than ABA (Hill et al., 1995). In seeds of western white pine (*Pinus monticola*), the accumulation level of 7′-hydroxylation ABA was high during the wet–cold process, while it peaked in the early stages of germination (Feurtado et al., 2004). The 9′-hydroxylation pathway is highly similar to the 8′-hydroxylation pathway, with the two products occurring under different environmental conditions. Both hydroxylation reactions are catalyzed by CYP707As, but the activity for catalyzing 9′-hydroxylation is very low, leading to the common belief that 9′-hydroxylation is a side reaction of 8′-hydroxylation (Okamoto et al., 2011; Chen K. et al., 2020). Similar to 8′-hydroxylation, 9′-hydroxylation produces neoPA through a spontaneous rearrangement reaction. NeoPA can be detected in immature seeds but is absent in seeds at later developmental stages, indicating that it may be metabolized during seed development (Zhou et al., 2004). A recent study utilizing liquid chromatography–mass spectrometry (LC-MS) techniques has discovered new enzymes involved in the 9′-hydroxylation metabolic pathway and its downstream metabolites. The Feng group identified the structure of the newly discovered neoPA metabolite in *Arabidopsis* using mass spectrometry analysis, isotope tracing, and chemical synthesis and named it epi-neoDPA. Furthermore, they identified NeoPAR1 (neoPA reductase 1) as the enzyme responsible for converting neoPA to epi-neoDPA. Phenotypic analysis of a *NeoPAR1* transgenic line suggested that *NeoPAR1* may play a regulatory role in *Arabidopsis* seed germination (Bai et al., 2022).

CYP707A is a key enzyme in ABA metabolism, with its gene expression in guard cells and vascular tissues controlling ABA metabolism and function under various conditions (Okamoto et al., 2009). *CYP707A*s are encoded by a multigene family in higher plant species (Hanada et al., 2011). Each member of this family exhibits distinct spatiotemporal expression patterns, indicating that the products of each gene may play roles in different physiological or developmental stages (Okamoto et al., 2006, 2009). The genes encoding CYP707A could be transcriptionally activated during seed germination and other developmental processes, as well as under stress conditions (Kushiro et al., 2004). In *Arabidopsis*, there are four *CYP707A* genes (*CYP707A1*, *CYP707A2*, *CYP707A3*, and *CYP707A4*). Among these, the *CYP707A1* and *CYP707A3* genes were highly expressed in most *Arabidopsis* tissues (Saito et al., 2004). They play important roles in ABA catabolism during the mid to late stages of seed development and post-germination. Conversely, *CYP707A2* was crucial during the late stages of seed development and germination (Okamoto et al., 2006; Umezawa et al., 2006; Zhu et al., 2011). The expression of all four *CYP707A* genes was induced by dehydration stress and subsequent rehydration, indicating that the ABA content of plants was maintained through a balance between biosynthesis and catabolism, including dehydration and rehydration feedback-induced catabolism (Umezawa et al., 2006).

In addition to *Arabidopsis*, the *CYP707A* gene family contains 10 genes in soybean. Structural and phylogenetic analyses suggested that these *GmCYP707A* genes were highly conserved with *CYP707A* genes in other monocotyledonous and dicotyledonous plants. Most of these genes were expressed in multiple soybean tissues and were induced by dehydration, rehydration, and salt stress (Zheng et al., 2012). In maize, *ZmCYP707A* genes were expressed in all tissues with highly tissue-specific and partially overlapping expression patterns. Among them, *ZmCYP707A1a* and *ZmCYP707A1b* were the major transcriptional components regulating ABA catabolism in the roots under drought stress (Vallabhaneni and Wurtzel, 2010). In addition to their role under stress conditions, *CYP707A*s also played crucial roles in other physiological processes. In tomato, *SlCYP707A2* was a critical gene regulating ABA catabolism, acting as a negative regulator in the fruit ripening process (Ji et al., 2014). Similarly, in sweet cherry, *PacCYP707A2* negatively regulated fruit ripening, while *PacCYP707A1* modulated ABA levels in response to dehydration during fruit development (Li et al., 2015). In pepper, *CYP707A*s (*CaCYP707A1*, *2*, *3*, and *4*) were significant for ABA degradation mediating the hydroxylation of ABA and influencing pollen development. The evidence indicated a potential mechanism by which ABA regulates pollen development (Kim et al., 2020). The barley *CYP707* homolog (*HvABA 8′-hydroxylation*) exhibited differential expression at various stages. Its expression level in the embryos of non-dormant grains was significantly higher than in dormant grains. *In situ* hybridization studies have shown that *HvABA 8′-hydroxylation* was more strongly expressed in the embryos of non-dormant grains (Millar et al., 2006).

In addition to the hydroxylation catabolic pathway, there is also a glycosylation pathway. The hydroxyl group at the C-1 position of the ABA molecule can bind with different chemical substances to form various conjugates, leading to inactivation. As previously mentioned, the inactive conjugate form ABA-GE is produced by ABA-uridine diphosphate (UDP) glucosyltransferases (UGTs) and is the major conjugate in the glycosylation catabolic pathway. ABA-GE is formed in the cytoplasm and stored in vacuoles. Through the interconversion between inactive ABA-GE and active ABA, the plant can maintain a stable ABA level, allowing it to better adapt to environmental changes (Priest et al., 2006; Chen T. T. et al., 2020).

UGTs belong to the glucosyltransferase (GT) family 1, which is the largest GT subgroup in the plant kingdom. In *Arabidopsis*, over 100 genes are known to encode UGTs, each containing a conserved sequence of 42 amino acids (Dietz et al., 2000; Ross et al., 2001). The gene encoding ABA-glucosyltransferase was first cloned from adzuki bean (*Vigna angularis*) and named *ABA O-glycosyl transferase* (*AOG*). The AOG protein catalyzes the conjugation of ABA with the glucose from UDP-glucose to form inactive ABA-GE. Its expression is regulated by environmental stress feedback, thereby modulating the levels of active ABA (Xu et al., 2002). Due to its broad substrate specificity, AOG from other species has rarely been identified (Wasilewska et al., 2008). Among the over 100 glucosyltransferase genes in *Arabidopsis*, eight have been identified as specific for (+)-ABA/(−)-ABA. Notably, UGT71B6, along with its closely related homologs UGT71B7 and UGT71B8, was crucial for maintaining the dynamic balance of ABA, which played a vital role in regulating ABA homeostasis and responding to various abiotic stresses, thus maintaining internal homeostasis in *Arabidopsis*. UGT71B6 exclusively recognized the naturally occurring (+)-ABA enantiomer and did not glucosylate the non-natural (−)-ABA enantiomer or the inactive 2-*trans*-ABA (Priest et al., 2006; Dong and Hwang, 2014; Dong et al., 2014). Subsequent studies have indicated that mutations in *ugt71c5* and low expression levels of *UGT71C5* in *Arabidopsis* resulted in delayed seed germination and enhanced drought tolerance. Conversely, overexpression of *UGT71C5* accelerated seed germination while reducing drought resistance (Liu et al., 2015). Under drought stress, ABA receptors RCAR12/13 could enhance the activity of UGT71C5, leading to the glucosylation of excess ABA into ABA-GE, which served as a rapid regulatory mechanism for plant growth and development (Ma et al., 2021). Overexpression of the putative glucosyltransferase gene *UGT85E1* in rice enhanced the drought tolerance of transgenic rice plants, whereas mutants of *ugt85e1* showed increased sensitivity to drought stress. It indicated the significant role of *UGT85E1* in mediating plant responses to drought and oxidative stress (Liu et al., 2021).

## 4 ABA receptors

Among all receptors, the PYR/Pyrabatin resistance like (PYL)/RCAR is the most extensively studied, named after pyrabactin, a selective ABA agonist synthesized in plants and localized in the cytoplasm and nucleus. The Park group initially used pyrabactin to screen 12 pyrabactin-resistant mutants from an *Arabidopsis* mutant library and named them *PYR1*. Through positional cloning, it was discovered that *PYR1* encoded a member of the cyclase subfamily within the START domain superfamily. It was also discovered that PYR1 binds to (+)-ABA. The PYR/PYLs bound to and inhibited PP2Cs in response to (+)-ABA. The PYR/PYLs controlled ligand-induced PP2C interaction, placing them at the top of a negative regulatory pathway that regulated ABA signal transduction by inhibiting PP2Cs (Park et al., 2009). In the same year, the Grill group identified the *ABI2* gene through positional cloning and homology to ABI1. They then used the yeast two-hybrid technique to screen for a protein belonging to the START superfamily, which they named RCAR1. RCAR1 could bind ABA, mediate ABA-dependent inactivation of ABI1 or ABI2, and antagonize the function of PP2C in plants. Other RCARs also mediated ABA-dependent regulation of ABI1 and ABI2, consistent with the combinatorial assembly of receptor complexes (Rodriguez et al., 1998; Ma et al., 2009). Subsequent research confirmed that RCARs and PYR/PYLs belong to the same family, differing only in nomenclature.

**Figure 4:**
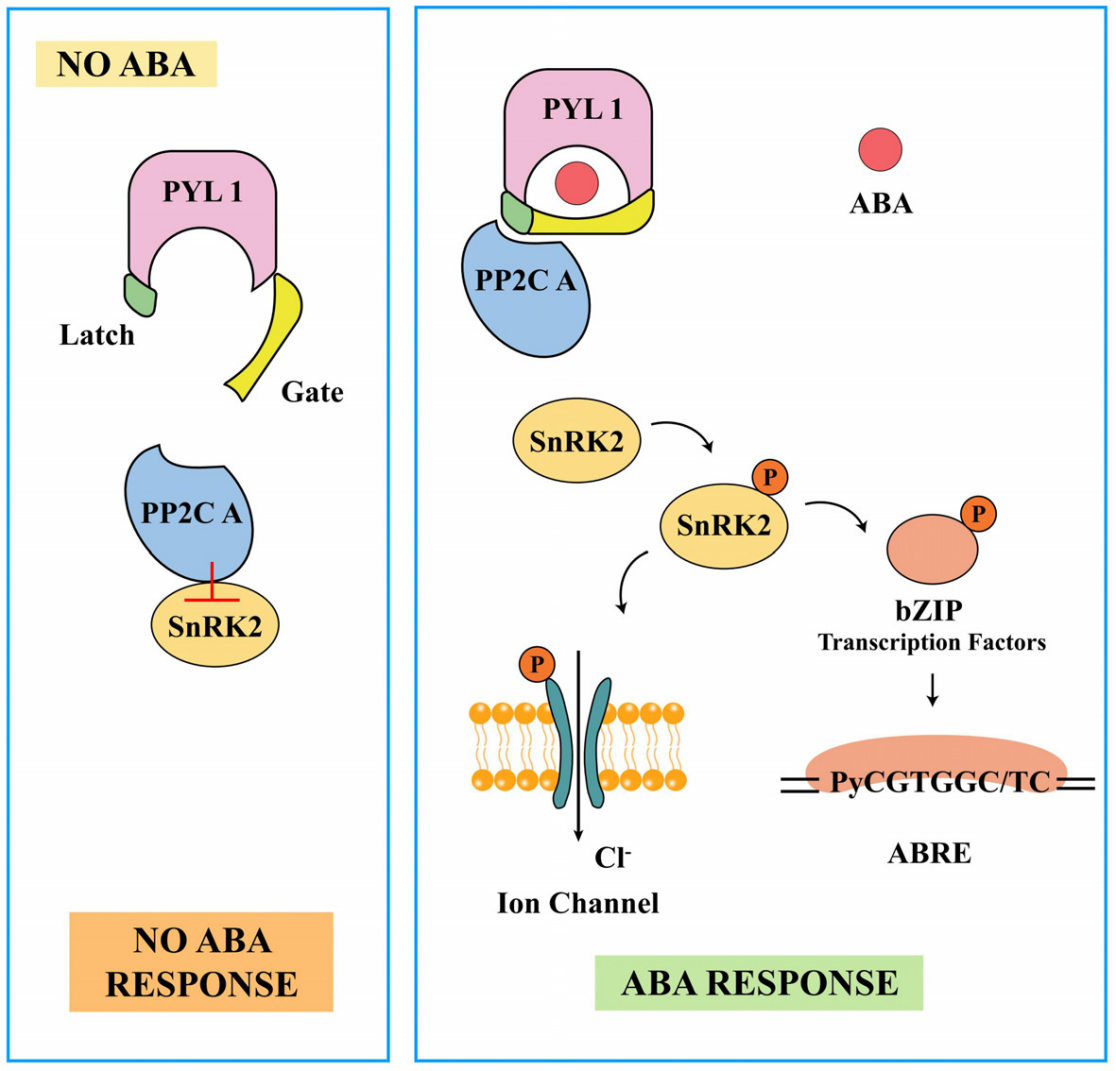
The abscisic acid (ABA)-dependent ABA receptor “Gate-Latch-Lock” model. The “Gate-Latch-Lock” model elegantly describes how ABA binding induces structural changes in its receptor, facilitating precise regulation of the ABA signaling pathway. Gate: In the absence of ABA, the receptor’s gate remains closed, preventing interaction with downstream signaling components. This gate is maintained in a closed position by the structural configuration of the receptor. Latch: When ABA binds to the receptor, it acts like a key that releases the latch. The binding of ABA induces a conformational change in the receptor, causing the latch to release and allowing the gate to open. This conformational change is essential for the activation of the receptor. Lock: Once the gate is open and the latch is released, the receptor can interact with downstream proteins, such as protein phosphatases. The binding of ABA effectively “locks” the receptor into an active conformation, ensuring that the signaling pathway remains activated as long as ABA is present.

By analyzing the crystal structures of the PYR1, PYL1, PYL2, PYL3, PYL5, PYL9, PYL10, and PYL13 proteins, it was found that the PYR/PYL/RACR family possesses a highly similar and conserved “helix-grip” fold with a hydrophobic pocket structure (Iyer et al., 2001; Santiago et al., 2009a, 2009b; Hao et al., 2011; Nakagawa et al., 2014). Initial studies have indicated that PYL1 used the specific ligand-binding site of START proteins to bind (+)-ABA, forming a hydrophobic pocket on its closed lid surface. The PYL1 bound to (+)-ABA acts like a plug, covering the active site of ABI1 with its hydrophobic pocket, leading to a tight interaction with the PP2C domain of ABI1 (Miyazono et al., 2009). Subsequent experiments have revealed that ABA receptors were regulated in an ABA-dependent manner through a mechanism known as the “Gate-Latch-Lock” mechanism. The term “apo-PYR/PYL/RACR receptor” refers to the state of PYR/PYL/RACR receptors when they were not bound to a ligand, containing an open ligand-binding pocket with gates on either side. These gates close in response to ABA through conformational changes in two highly conserved β-loops (acting as the gate and the latch) (Melcher et al., 2009) (**Figure 4**). When not bound to ABA, the ligand-binding pocket remains in an open state. Upon ABA binding, it displaces the serine residues on the “gate” loop outward, while the proline residues on the “gate” loop shift to close the cavity. The histidine residues on the “latch” loop then reorient their imidazole rings into the cavity through van der Waals interactions with the ABA cyclohexene ring, altering the conformation of the “latch” loop and locking the “gate” loop closed through hydrogen bonding and van der Waals forces (Santiago et al., 2009a; Yin et al., 2009; Santiago et al., 2012; Miyakawa et al., 2013). The closure of the gate induced by ABA creates a surface that allows the receptor to dock and competitively inhibit the PP2C active site. A conserved tryptophan in PP2C directly inserts between the gate and the lock, further locking the overall structure in a closed conformation and inhibiting the protein phosphatase activity of PP2C by blocking substrates from entering the catalytic center (Santiago et al., 2012). In addition, within the ABA ligand-binding pocket, ABA enhances their binding with PYR/PYLs/RACRs through a combination of ionic bonds, hydrophobic interactions, and water-mediated hydrogen bonding (Zhang et al., 2015).

PYR/PYL/RCAR proteins can be classified into two distinct subclasses based on the oligomeric state of their apo forms: monomeric PYLs and dimeric PYLs (Dupeux et al., 2011). Subsequent studies have discovered that a subclass of PYLs, exemplified by PYL10, was capable of inhibiting PP2C without any ligand. Crystal structure analyses of PYL10, both in its apo form and when bound to PP2C, revealed that ABA-independent inhibition of PP2C necessitated the monomeric state of PYLs (Hao et al., 2011; Li et al., 2013). Subsequently, another research team identified a third subclass, PYL3, which existed in both monomeric and dimeric states. Upon ABA binding, PYL3 significantly promoted the formation of monomeric PYL3, thereby greatly enhancing the efficiency of PP2C inhibition (Zhang et al., 2012a, 2012b). Studies on PYL3 have investigated the impact of the oligomeric state of PYLs on their interaction with PP2C. When PYL3 was in the apo state and upon ABA binding, there were two significant structural differences: the loop on the side of the ABA binding pocket changed, and the C-terminal α-helix underwent a transformation. These conformational changes not only stabilized the binding of ABA to PYL3 but also created a new surface that facilitated PP2C binding (Zhang et al., 2012b). From these studies, it was found that dimeric PYLs had a weak ability to bind PP2Cs and had to rely on the presence of ABA. In contrast, monomeric PYLs could directly inhibit the phosphatase activity of PP2Cs without depending on ABA. Monomeric PYLs also interacted with PP2Cs, but this interaction was significantly enhanced in the presence of ABA.

In the *Arabidopsis*, there are 14 members of the PYR1 and PYL family, designated as PYL1–13/RCAR1–RCAR14. Functionally, PYL proteins exhibit redundancy in plants. Single mutants of PYLs generally show no significant phenotypic differences compared to the wild type and exhibit unchanged sensitivity to ABA. However, they may display certain defects in seed germination, root growth, and stomatal closure. Conversely, multiple mutant variants of PYLs show noticeable differences from the wild type. For instance, triple mutants such as *pyr1/pyl1/pyl4* and quadruple mutants like *pyr1/pyl1/pyl2/pyl4* demonstrated strong insensitivity to ABA (Park et al., 2009). These pieces of evidence collectively suggested functional redundancy among these ABA receptors. Overexpression of *RCAR1/PYL9*, *PYL5/RCAR8*, or *PYL8/RCAR3* enhanced the ABA response in *Arabidopsis* and conferred increased drought tolerance (Ma et al., 2009; Park et al., 2009). Recombinant analysis on *Arabidopsis* protoplasts revealed that all PYLs except PYL13 could bind with ABA, subsequently inhibiting PP2C activity (Fujii et al., 2009; Li et al., 2013). Consistently, the key lysine residues involved in ABA binding among the other 13 members were not conserved in PYL13 (Miyazono et al., 2009; Yin et al., 2009). Subsequent research has indicated that although PYL13 was not an ABA receptor, it could interact with both PYL receptors and PP2Cs as a co-receptor, thereby inhibiting both pathways involved in ABA regulation (Zhao et al., 2013).

PYR/PYL/RCARs participate in various crucial physiological activities within plants through the ABA signaling pathway. As ABA receptors, overexpression of PYLs significantly enhances plant sensitivity to ABA, thereby reinforcing ABA-induced effects such as seed dormancy promotion and inhibition of plant growth (Miyakawa et al., 2013). The nucleotide sequences of *Arabidopsis PYL8* and *PYL9* exhibited high similarity. Notably, single mutants of *pyl8* displayed a significantly shorter lateral root quiescent period compared to double mutants of *pyl8*/*pyl9*. Moreover, the concentration of IAA required for the restoration of lateral root growth is slightly higher in single mutants, indicating functional redundancy in the regulation of lateral root elongation between the *PYL8* and *PYL9* genes (Zhao et al., 2014). In addition, overexpression of *PtPYL4* in *Populus trichocarpa* enhanced the activity of antioxidant enzymes and the content of ascorbic acid, thereby accelerating the scavenging of reactive oxygen species (Li Q. et al., 2023). Constitutive expression of *PtPYRL1* and *PtPYRL5* positively regulated ABA signaling, significantly enhancing resistance to drought, osmotic, and cold stresses, including increased stomatal closure and reduced leaf dehydration (Yu et al., 2017). Based on gene expression and protein domain analysis, it was inferred that *PaPYL9* may be involved in ABA signaling and apricot fruit ripening (Jia M.X. et al., 2022). Similarly, *SlPYL9* in tomato regulated flower abscission and fruit ripening (Kai et al., 2019). Overexpression of the *VaPYL4* gene enhanced cold tolerance in grape *Vitis americana* callus tissue and also increased resistance to drought and salt stresses in *Arabidopsis* (Ren et al., 2022). Tomato plants with silenced *SlPYL4* wilted faster under drought conditions compared to control plants (Li et al., 2022). Silencing of *VvPYL4* impairs resistance to *Plasmopara viticola*, and experimental evidence suggested that this was related to the suppression of genes involved in the ABA signaling pathway (Liu L. et al., 2020). The latest research provides insights into previously unknown negative regulatory mechanisms of the ABA signaling pathway. Under drought stress, ABA inhibited the phosphorylation of PYL through CIPK (CBL-interacting protein kinase) 1, allowing plants to respond to ABA signaling and survive in unfavorable environments. This signaling pathway is mediated by CBL1/9-CIPK1-PYL, making plants more sensitive to drought stress (You et al., 2023).

## 5 The signaling pathway of ABA

The ABA signaling cascade is initiated when ABA receptors bind to their target cells or receptors, triggering downstream responses. The key components of ABA signal transduction include PYR/PYL/RCAR (receptors), PP2C (negative regulators), and Sucrose Non-fermenting Related Protein Kinase 2 (SnRK2; positive regulators). These elements work together to mediate plant defense mechanisms against various stressors (Nakashima and Yamaguchi-Shinozaki, 2013). In the presence of ABA, PYR/PYL/RCAR forms a complex structure with PP2C, inhibiting the negative regulation of PP2C and further activating the downstream protein phosphorylation function of the positive regulator SnRK2. This includes transcription factors that promote the expression of ABA-responsive genes (Yoshida et al., 2014). Phosphorylation and dephosphorylation events determine the activation of SnRK2, thereby regulating ABA signal transduction. Under normal conditions, the interaction between PP2Cs and C-terminal subdomain II of SnRK2s suppresses ABA signaling and dephosphorylates them, thereby promoting appropriate plant growth. Conversely, under stress conditions, PYR1 and PYLs interact with PP2Cs. This disruption leads to the release and activation of SnRK2s, as PP2Cs are unable to dephosphorylate the Ser/Thr residues in the activation loop of SnRK2s (Umezawa et al., 2009; Szostkiewicz et al., 2010; Antoni et al., 2012). PYLs exhibit two distinct binding modes, known as the ABA-enhanced and ABA-dependent discrete binding models, each with different characteristics regarding their interactions with ABA and PP2Cs (Hao et al., 2011). The mechanism described above represents the widely accepted and recognized signaling pathway of ABA, where SnRK2 serves as a core component coupled with ABA receptors in the signaling cascade.

However, signal transduction is an exceedingly complex network, and the latest research has uncovered the involvement of Rapidly Accelerated Fibrosarcoma-like Protein Kinases (Rafs) in ABA-triggered SnRK2 activation. The B2 and B3 Raf subgroups phosphorylate Ser171 and Ser175 of SnRK2.6 with varying specificities, and this *trans*-phosphorylation is essential for initiating SnRK2.6 activation. Once phosphorylated by Raf, SnRK2.6 undergoes rapid intermolecular autophosphorylation, further activating additional SnRK2 proteins and amplifying the response. ABA did not activate Raf in the B2 and B3 subgroups; instead, the basal level of Raf kinase activity was crucial for initiating SnRK2 activation and ABA signal transduction. This research highlights the crucial RAF-SnRK2 cascade in ABA receptor-coupled core signal transduction and a unique activation mechanism for initiating and amplifying stress signal transduction in higher plants (Lin et al., 2021). The study presented a novel description of the plant ABA receptor-coupled signaling pathway: ABA-PYL-PP2C regulated the binding inhibition or release of SnRK2, while RAF facilitates self-activation of SnRK2 through phosphorylation, initiating the kinase activation process for SnRK2. This mechanism is likely to be widespread and crucial in protein kinase-coupled pathways, enabling rapid responses to environmental stressors.

According to their biochemical and structural properties, protein phosphatases are classified into the PP1, PP2A, and PP2C groups. Genetic evidence indicates that both PP2A and PP2C are involved in ABA signal transduction (Shenolikar, 1994; Jung et al., 2020). *abi1* and *abi2* genes were initially identified as ABA-insensitive mutants and later found to encode PP2Cs (Leung et al., 1994; Meyer et al., 1994). The *abi1-1* mutation, which converted Gly-180 of the PP2C domain to Asp, significantly reduced the enzymatic activity of ABI1 (Bertauche et al., 1996). Similarly, the *abi2-1* mutation, which replaced Gly-168 with Asp, also markedly decreased the enzyme activity (Leung et al., 1997). Both *abi1-1* and *abi2-1* were dominant mutations and confer ABA insensitivity in different tissues and developmental stages. Homologous *ABIl* and *ABI2* exhibit partial functional redundancy in ABA signaling, providing a mechanism for maintaining ABA homeostasis in plants (Leung et al., 1997). In *Arabidopsis*, 76 genes encode PP2C proteins, with 70 of these genes categorized into 10 groups (A–J), with group A containing the majority of the identified genes related to ABA signal transduction, including *ABI1* and *ABI2* (Schweighofer et al., 2004). A-group PP2Cs exhibit functional redundancy at the molecular level. Mutants with double or triple mutations in PP2Cs showed enhanced ABA hypersensitivity. However, they also have unique roles in different tissues and organs (Nishimura et al., 2007).

PP2C plays a crucial role in both normal plant growth and under stress conditions; different groups of PP2Cs have distinct functions (Jung et al., 2020). In the context of plants responding to abiotic stress through PP2C, a recent study has proposed a signaling pathway under drought stress. The activity of Casein Kinase-like kinase 2 (CKL2) was released by forming a complex containing ABA, PP2C, and PYR1/PYL/RCAR receptors. ABA signaling regulates actin filament reorganization to maintain stomatal closure during drought stress. The feedback regulation of CKL2 on PYL1, ABI1, and ABI2 may fine-tune ABA signaling and influence the plant ABA response (Shi et al., 2022). In *Arabidopsis*, AIP1 is a key PP2C involved in ABA-mediated inhibition of germination under cold stress conditions. This protein functions downstream of RCAR5/PYL11 and regulates seed germination by interacting with RCAR5/PYL11 in the presence of ABA (Lim and Lee, 2021). In maize, 102 *ZmPP2C* genes have been identified, and it has been found that *ZmPP2C*s were highly conserved throughout evolution, playing key roles in various abiotic stress responses (He et al., 2019). Among them, *ZmPP2C15* positively regulates drought tolerance in maize by influencing antioxidant enzyme activities and the content of osmotic regulatory substances (Pang et al., 2024). Conversely, ZmPP84, a group F PP2C, negatively regulates drought tolerance by dephosphorylating ZmMEK1 (a MAPKK family member), inhibiting stomatal closure (Guo et al., 2023). In tomato, the expression of *SlPP2C3* was induced by ABA and negatively correlated with fruit ripening. *SlPP2C3* plays an important role in regulating tomato fruit ripening and fruit glossiness (Liang et al., 2021). In rice, the OsPP2C09 protein interacted with core components of the ABA signaling pathway and exhibited PP2C phosphatase activity *in vitro*, positively influencing plant growth but acting as a negative regulator of drought tolerance through ABA signaling (Miao et al., 2020). OsPP2C08 negatively regulates ABA signaling and positively regulates GA signaling (Song et al., 2023).

The PP2A signaling branch operates parallel to the typical PYL-PP2C mechanism (Pernas et al., 2007). PP2A consists of three subunits, and five genes encoding catalytic subunit isoforms have been identified in *Arabidopsis*, along with three genes encoding scaffold subunit A and at least 16 encoding regulatory subunit B. The extensive isoforms of each subunit contribute to PP2A’s multifunctionality, but this complexity also makes its regulation less understood compared to PP2C (Terol et al., 2002). In *Arabidopsis*, the expression of the PP2A gene *RCN1* was essential for ABA-induced stomatal closure and the activation of slow anion channels by ABA. Moreover, mutations in *rcn1* result in reduced ABA sensitivity, affecting seed germination and ABA-responsive gene expression (Kwak et al., 2002). Pernas et al. firmly established a specific catalytic subunit, PP2Ac-2, as a component of the signaling pathway that inhibited ABA responses. Loss-of-function mutations in *pp2ac-2* render plants highly sensitive to ABA, altering responses to developmental and environmental processes mediated by ABA, such as primary and lateral root development, seed germination, and responses to drought, high salt, and sugar stresses. Conversely, plants overexpressing *PP2Ac-2* exhibited reduced sensitivity to ABA compared to the wild type (Pernas et al., 2007). A study has found that PYL-PP2A signaling regulated root gravitropism and lateral root formation by modulating auxin transport in plants. Under optimal conditions, PYL ABA receptors interacted with the catalytic subunit of PP2A, increasing its phosphatase activity to counteract the phosphorylation of Pin-Formed (PIN) auxin transport proteins mediated by PINOID (PID) kinase (Li et al., 2020).

SnRK2s belong to the Sucrose Non-fermenting-1 (SNF1)-related protein kinase family, which are a type of serine/threonine protein kinases. The first SnRK2 cDNA clone (*PKABA1*) was identified by hybridization screening of an ABA-treated wheat embryo cDNA library using a probe synthesized from conserved kinase domain segments (Anderberg and Walker-Simmons, 1992). In *Arabidopsis*, the SnRK2 family comprises 10 members, SnRK2.1–SnRK2.10 (or SRK2A–SRK2J) (Hrabak et al., 2003; Saha et al., 2014), which are divided into three subclasses (I, II, and III) (Hubbard et al., 2010). Among them, three members, SnRK2.2, SnRK2.3, and SnRK2.6, were rapidly activated within minutes following exogenous ABA application. All SnRK2s, except SnRK2.9, were activated by osmotic stress (Boudsocq et al., 2004; Fujii and Zhu, 2009; Fujita et al., 2009; Fujii et al., 2011). SnRK2.6, also known as open stomata 1 (OST1), was primarily expressed in guard cells, whereas SnRK2.2 and SnRK2.3 were ubiquitously expressed (Mustilli et al., 2002; Fujii et al., 2007; Li M.M. et al., 2023). Comprehensive analysis of SnRK2 in *Arabidopsis* and wheat indicated that these three subclasses exhibited distinct activation patterns in response to ABA and osmotic stress (Saha et al., 2014; Zhang et al., 2016; Jiang et al., 2022). They are involved in responses to various stresses, including high salinity, cold, and PEG-induced osmotic stress. SnRK2s in subclass III are strongly induced by ABA, while those in subclass II show a weaker response to ABA compared to subclass III, and subclass I SnRK2s are not activated by ABA treatment. Subclass III comprises three kinases, namely, SRK2D/SnRK2.2, SRK2E/OST1/SnRK2.6, and SRK2I/SnRK2.3, which participate in ABA-regulated stress responses. In contrast, SnRK2s in subclasses I and II respond to various abiotic stresses in an ABA-independent manner. All SnRK2s play crucial roles in the initiation of ABA (Boudsocq et al., 2007; Saha et al., 2014; Zhang et al., 2016; Jiang et al., 2022) signaling. New evidence indicated that SnRK2.4 was a crucial regulator of aquaporins and root hydraulics (Shahzad et al., 2024), and SnRK2.10 was involved in regulating reactive oxygen species (ROS) homeostasis in response to salinity in *Arabidopsis* (Szymańska et al., 2019).

SnRK2s are positive regulators in the ABA signaling network, capable of activating downstream signaling pathways. Ongoing research has provided valuable insights and potential genetic resources for enhancing plant environmental adaptability. In various plant species, SnRK2s contribute significantly to stress responses, including GmSnRKs in soybeans (Shen et al., 2022) and ZmSnRK2.9 in maize, which are crucial for drought tolerance (Yan et al., 2024) (Zhao et al., 2017), and SAPK9 in rice, which is particularly responsive during the reproductive stage (Dey et al., 2016). From this, we can infer that SnRKs play a central role in the ABA-mediated stress signaling pathway in plants.

Subclass III SnRK2s are believed to undergo self-activation through autophosphorylation following release from PP2C-mediated inhibition. However, recent reports suggested that specific protein kinases could phosphorylate and activate these subclass III SnRK2s (Hasan et al., 2022). Therefore, under stress conditions, are SnRKs activated either through self-activation or by external activation to initiate defense mechanisms? The upstream components required for this process remain unknown, and the molecular mechanisms involved are currently not well understood. Reports indicated that the B2, B3, and B4 subfamilies played crucial roles in early osmotic stress and ABA signal transduction in *Arabidopsis*. Raf-B2, Raf-B3, and Raf-B4 were rapidly activated by osmotic pressure and were essential for the phosphorylation and activation of SnRK2 (Lin et al., 2020). Further research revealed that when PYL receptors were released from PP2C-mediated inhibition upon binding with ABA, B2-Raf remained active continuously and activated SnRK2, whereas B3-RAF activated SnRK2 in an ABA-independent manner only under stress conditions, thereby enhancing SnRK2 activity. Autophosphorylation of subclass III SnRK2s alone was insufficient to respond to ABA signaling; hence, B2-Raf was required to activate SnRK2 in an ABA-dependent manner. Experimental findings suggested that B2-Raf regulated subclass III SnRK2 during the early stages of drought stress, while B3-RAF regulated SnRK2 during the later stages of drought stress. This indicated that B2-Raf was an essential kinase for responding to ABA and activating subclass III SnRK2 under mild osmotic stress conditions, whereas B3-Raf acted as an enhancer of SnRK2 activity under severe stress conditions (Soma et al., 2023). It was also reported that SnRK2 was activated by the ARK (a kind of Rafs), representing a primary mechanism for ABA and abiotic stress responses (Islam et al., 2021). The signaling pathway of the plant immune receptor activated by the hormone ABA has also seen recent advancements.

Plant nucleotide-binding, leucine-rich repeat immune receptor (NLR) Sw-5b mimicked the behavior of ABA receptors and directly utilized the ABA central regulatory factor PP2C-SnRK2 complex to activate ABA-dependent defense against viral pathogens (Huang et al., 2024). Additionally, there existed a rapid, largely unknown, ABA-independent osmotic stress SnRK2 signaling pathway activation. It has been identified that functionally redundant mitogen-activated protein kinase kinases (M3Ks) were essential for activating SnRK2 kinases. Plants with triple knockouts of M3Ks exhibited reduced ABA sensitivity and severely impaired rapid osmotic stress-induced SnRK2 activation. These findings suggest that this evolutionary branch of M3Ks was necessary for the activation of SnRK2 kinases in both ABA and osmotic stress responses, thereby facilitating robust ABA and osmotic stress signal transduction (Takahashi et al., 2020).

Two downstream targets of two subclasses of SnRK2s have been identified. One class comprises bZIP domain transcription factors, which serve as key transcription factors in the ABA signaling pathway. The bZIP transcription factor family proteins consist of three conserved domains at the N-terminus (C1, C2, and C3) and a conserved basic leucine zipper structure at the C-terminus (Uno et al., 2000). These transcription factors contain a conserved hydrophobic leucine zipper structure formed by seven amino acids, which can bind to a conserved ABA-responsive *cis*-acting element (*PyCGTGGC/TC*) in the promoter region of ABA-induced genes, thereby activating downstream gene expression. ABRE (ABA Responsive Element) include AREB (ABA Responsive Element Binding Protein), ABRE-Binding Factors (ABFs), and ABI5 (Choi et al., 2000; Lopez-Molina and Chua, 2000; Uno et al., 2000; Bensmihen et al., 2002). ABRE, as a *cis*-acting DNA element, regulates gene expression in response to ABA. It was initially discovered in the wheat gene *Em* and primarily functions in seeds during the late stages of embryogenesis (Guiltinan et al., 1990). In *Arabidopsis*, there are nine ARBEs, including ABF1, AREB1/ABF2, ABF3, AREB2/ABF4, and ABI5, which are regulated by genes such as ABI3 or ABI4 (Fujita et al., 2005). Among them, AREB1, AREB2, and ABF3 act as master transcription factors, showing significant functional overlap. They all require ABA for full activation and can form hetero- or homo-dimers to function in the nucleus. They also can interact with SRK2D/SnRK2.2 (a member of the SnRK family and a regulator of AREB1) (Yoshida et al., 2010). A systematic analysis of the AREB/ABF transcription factor family in wheat revealed that most *TaABF* genes in the A and B groups were highly expressed in various tissues and upregulated after exposure to abiotic stresses such as drought, low temperature, and low nitrogen. Additionally, some *TaABF* genes in the C group exhibited specific expression in grain tissues (Yang et al., 2024). In tomatoes, *AREB/ABF* genes were divided into two groups. Analysis of *cis*-acting elements suggested that most of these *AREB/ABF* genes contained multiple hormone and stress-related elements (Pan et al., 2023). New evidence found that ABA significantly induced the expression of *AREB/ABF* genes and the accumulation of endogenous ABF proteins. Moreover, ABFs themselves were involved in this induction process, thus providing another mode of ABA regulation for ABF proteins, in addition to the ABA-induced phosphorylation by SnRK2 protein kinases. In other words, ABFs mediated the rapid ABA induction of group A *PP2C* genes, thereby playing a role in the negative feedback regulation of ABA signaling (Wang et al., 2019). In addition to the aforementioned *cis*-acting elements, a similar *cis*-acting element known as the G-box (CACGTGGC) was identified in the promoters of light-regulated genes such as *RbcS* in *Arabidopsis*. This element encodes the ribulose-1,5-bisphosphate carboxylase/oxygenase (Rubisco) complex (Menkens et al., 1995). Deletion and mutation analyses of promoters demonstrated that a single copy of ABRE or G-box was crucial but not entirely sufficient for ABA-induced gene expression. They also required an additional promoter element called a coupling element (CE) to properly respond to ABA transcriptional activation (Shen et al., 2004). For instance, in *Arabidopsis*, two *ABRE*s were found in the *RD29B* promoter, serving as *cis*-regulatory elements in ABA-dependent expression (Uno et al., 2000).

Another class of targets includes two ion channels, namely, Slow Anion Channel 1 (SLAC1) and inwardly rectifying K+ channel (KAT), as well as NADPH oxidases (Vahisalu et al., 2008). SnRK2.6 (OST1) interacted with SLAC1 and ABI1. When ABI1 was integrated into the SLAC1/OST1 complex, it inhibited SLAC1 activation, thereby regulating stomatal movement. This indicates that SLAC1 was controlled by OST1/ABI1-dependent phosphorylation/dephosphorylation, which was a calcium-independent activation mechanism (Geiger et al., 2009; Lee et al., 2009). SLAC1 could also be activated by calcium-dependent kinases (CPKs) 21 and 23, which were inhibited by ABI1 (Geiger et al., 2010). CPK21 and CPK23 not only activated SLAC1 in the presence of ABA but also activated its homolog SLAC3 (Geiger et al., 2011). Phosphorylation mediated by SnRK2 activated SLAC1 and inhibited KAT, leading to plasma membrane depolarization and activation of outward K^+^ channels. Leakage of anions and K^+^ caused guard cell swelling loss, resulting in stomatal closure (Hubbard et al., 2010).

Therefore, the ABA signaling transduction process is a complex regulatory network with multiple elements involved in regulation. The interaction among different members of the PYR/PYL/RCAR, PP2C, and SnRK2 families may explain the complexity of ABA signal transduction, but it also increases the difficulty of studying the ABA regulatory network within plants.

## 6 The transport of ABA

When plant roots were placed in a solution containing exogenous ABA, a rapid increase in ABA concentration was measured in the leaves, which indicated the presence of an efficient ABA transport system within the plant, capable of transporting ABA to where it was needed (Kuromori et al., 2018). ABA in plants exists in two forms: the anionic form ABA− and the protonated form ABAH. ABAH exhibits weak acidity and its own hydrophobicity. Therefore, it is believed that the protonated form of ABA can easily pass through biological membranes without the need for specific protein-mediated passive diffusion mechanisms (Wilkinson and Davies, 2002). However, due to the differences in the environment inside and outside the plasma membrane, the transport of ABA out of the plasma membrane to specific locations requires mediation by transport proteins. ABA transport proteins that have been identified include ATP-binding cassette (ABC) transporter proteins (Kuromori et al., 2010), Nitrate Transporter 1/Peptide Transporter Family (NPF) (Kanno et al., 2012), and Detoxification Efflux Carrier/Multidrug and Toxic Compound Extrusion (DEX/MATE) transporters (Zhang et al., 2014; Merilo et al., 2015).

**Figure 5:**
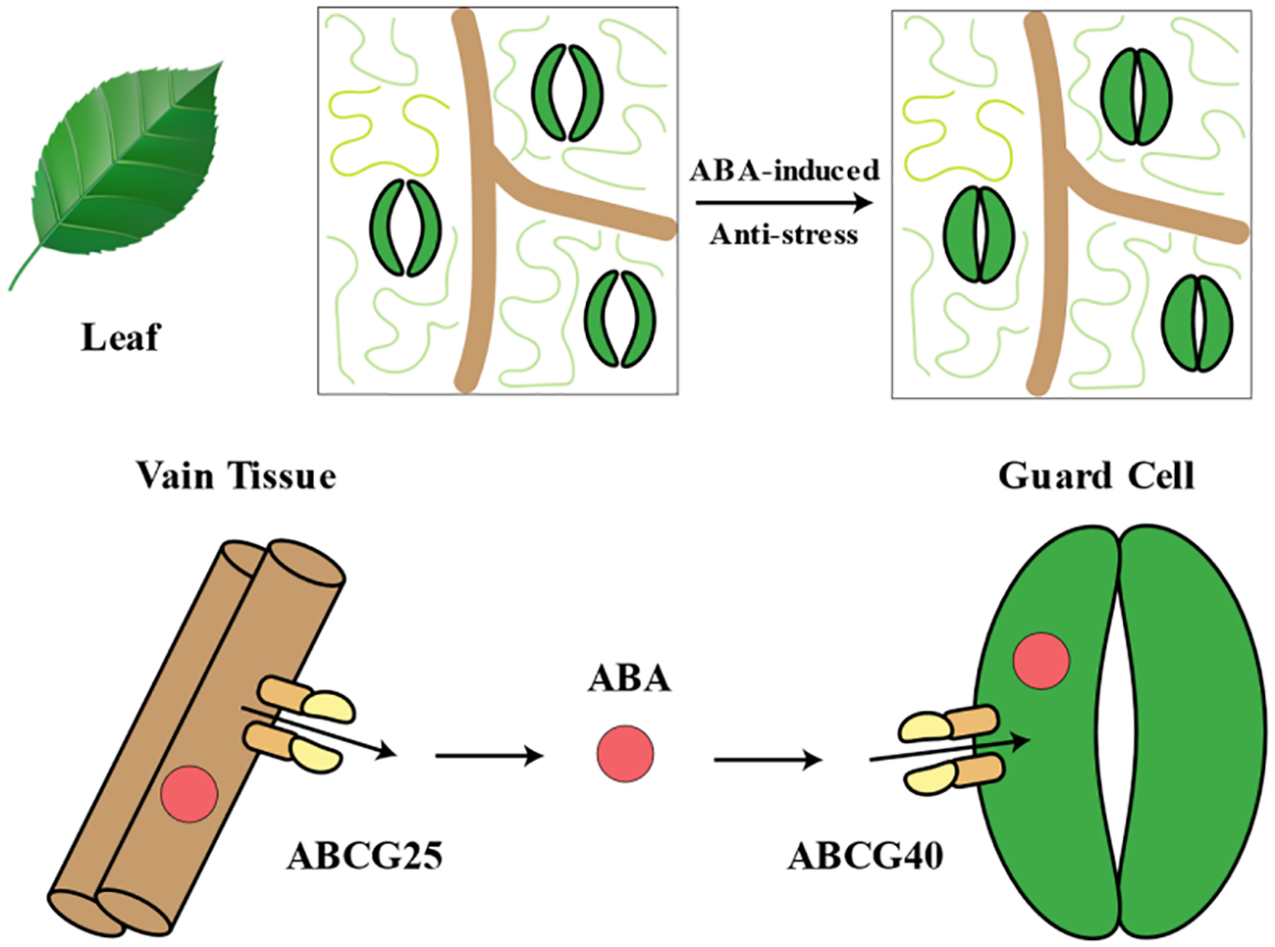
Abscisic acid (ABA) transport system in leaf. During stress conditions, ABCG25 and ABCG40, as key ABA transport proteins, play crucial roles. ABCG25 is responsible for transporting ABA from the vascular bundle to the extracellular region surrounding the guard cells. By transporting ABA to this area, ABCG25 facilitates subsequent uptake by the guard cells. ABCG40, however, is responsible for the uptake of ABA from the extracellular space into the guard cells. By absorbing ABA, ABCG40 promotes the accumulation of ABA within the guard cells. The concerted action of ABCG25 and ABCG40 transports ABA from the vascular bundle to the extracellular region surrounding the guard cells, ultimately leading to stomatal closure. Stomatal closure is a critical physiological response of plants to stress, as it helps conserve water by reducing transpiration.

ABC transporter proteins were initially isolated and cloned from *Arabidopsis* through genetic screens for ABA sensitivity mutants. The gene *AtABCG25*, encoding one of these proteins, was identified. *AtABCG25* was primarily expressed in vascular tissues and localized to the plasma membrane. Mutants lacking *AtABCG25* exhibited increased sensitivity to exogenous ABA, while overexpression lines showed reduced ABA sensitivity during post-germination growth and higher leaf temperatures, indicating its role in stomatal regulation. Biochemical analyses suggested that AtABCG25 mediated ATP-dependent ABA efflux. These findings strongly indicated that AtABCG25 acted as an ABA exporter and participated in intercellular ABA signaling pathways as a transporter protein (Kuromori et al., 2010). AtABCG40/AtPDR12 was responsible for uptaking ABA from the extracellular space, serving as a plasma membrane ABA uptake transporter protein. Biochemical analysis indicated that cells expressing *AtABCG40* showed increased absorption of ABA, while protoplasts from *atabcg40* mutants exhibited reduced ABA absorption. Mutants lacking functional AtABCG40 displayed slower stomatal responses to ABA, leading to decreased drought tolerance, suggesting the necessity of ABCG40 for timely ABA responsiveness (Kang et al., 2010). AtABCG25 mediated the export of ABA from the cell to the extracellular space, while AtABCG40 mediated the import of ABA from the extracellular space into the cell. The promoter activity of AtABCG25 was detected in vascular tissues, whereas the promoter activity of AtABCG40 was detected in guard cells (**Figure 5**). This observation suggested that ABA transport occurs from vascular tissues to guard cells. ABC transporter proteins comprise one of the largest protein families, subdivided into eight subfamilies, ABCA to ABCH, in eukaryotic organisms (Verrier et al., 2008). In *Arabidopsis*, there are approximately 129 ABA ABC transporter proteins, with many members of the ABCG subfamily believed to play a role in ABA transport (Kumar et al., 2022). In addition to the aforementioned AtABCG25 and AtABCG40, other ABCG transporter proteins also played crucial roles in ABA import and export. AtABCG27 served as an ABA transporter on the plasma membrane, with two ABA-responsive elements on its gene promoter, enabling the regulation of ovule and leaf development under normal, non-stress conditions (Kim et al., 2022). Four AtABCG transporter proteins cooperatively transported ABA from the endosperm to the embryo. Among them, AtABCG25 and AtABCG31 exported ABA from endosperm to multiple sites in the plant, while AtABCG30 and AtABCG40 imported ABA into the embryo, regulating seed germination (Kang et al., 2015). Mutant plants of *atabcg22* exhibited lower leaf temperatures and increased water loss and were more susceptible to drought stress compared to wild-type plants, indicating that AtABCG22 was involved in stomatal regulation through promoting ABA uptake. Its function was associated with guard cell responses in *Arabidopsis* (Kuromori et al., 2011).

*Arabidopsis* possesses 53 genes encoding NPF proteins. Initially identified as nitrate or dipeptide/tripeptide transporters, studies have indicated that NPF proteins can also transport plant hormones (Chiba et al., 2015; Corratgé-Faillie and Lacombe, 2017). At the outset of the investigation, it was found that the NPF protein AtNPF4.6 functioned as an ABA transporter. Interestingly, the ABA transport activity of AtNPF4.6 remained unaffected by excessive nitrate, and *npf4.6* mutants demonstrated reduced sensitivity to ABA during germination compared to wild-type plants (Kanno et al., 2013). Further investigation revealed that the stability of NPF4.6/NRT1.2 and its control over ABA input activity were precisely regulated through phosphorylation and degradation in response to environmental stress stimuli (Zhang et al., 2021). Subsequent detailed studies on NPF4.6, as well as NPF4.5, have revealed that the activity of both ABA transporter proteins depended on pH. Additionally, unlike ABA receptors, they did not exhibit competitive inhibition by ABA analogs, indicating distinct selectivity (Léran et al., 2020). The promoter activity of AIT1/NRT1.2 surrounding vascular tissues in inflorescence, stem, leaf, and root was examined. The data indicated that the function of AIT1/NRT1.2 as an inputter for ABA biosynthesis was crucial for regulating stomatal aperture in inflorescence stems (Kanno et al., 2012). When heterologously expressed, NPF5.1 mediated cellular ABA uptake. The leaf surface temperature was higher in *atnpf5.1* mutants. The expression of AtNPF5.1 was detected in several leaf cell types. It was also observed that AtNPF5.1 negatively regulated stomatal closure by modulating the amount of ABA transported from vascular tissues to guard cells (Shimizu et al., 2021). NPF4.2 and NPF4.7 were also identified as having the capability to transport ABA (Léran et al., 2020). In the latest study, it was found that plasma membrane-localized NPF2.12 and NPF2.13 promoted the long-distance transport of ABA from the phloem to the endodermis in roots after unloading. Meanwhile, vacuolar membrane-localized NPF2.14 facilitated the accumulation of ABA within the pericycle vacuoles, providing a hormone source for root lignification (Binenbaum et al., 2023).

DTX/MATE transporters are highly conserved across organisms ranging from bacteria to plants and animals (Omote et al., 2006). In the *Arabidopsis* genome, there are 58 members of the DTX/MATE family, several of which have been characterized as transporters for organic acids and secondary metabolites (Li et al., 2002). Notably, DTX50, DTX/MATE family number, was identified as an ABA efflux transporter, playing a pivotal role in ABA-mediated growth inhibition and response to drought stress. DTX50 was predominantly expressed in vascular tissues and guard cells, localized to the plasma membrane, and its expression was significantly upregulated by exogenous ABA (Zhang et al., 2014). Moreover, MATE45 is instrumental in modulating the level and distribution of ABA in both meristematic and non-meristematic tissues, crucial for growth regulation (Kovinich et al., 2018).

In the preceding discussion, the reciprocal conversion mechanism between ABA-GE and ABA, known to effectively maintain a relatively stable level of ABA within plant systems, was elaborated upon. Inactive ABA-GE also participated in long-distance ABA transport (Cutler and Krochko, 1999). ABA-GE formation occurred within the cytoplasm of root cells, with the endodermis and Casparian strip serving as ideal barriers to ABA-GE in root cells (Lehmann and Glund, 1986). However, in scenarios where the endodermis was absent (as seen in legumes and hydroponic plants), ABA-GE would be dragged into the extracellular space of root cortex cells. Within the extracellular space, β-glucosidase could hydrolyze the conjugate and release free ABA, subsequently transported via symplastic transport to the parenchyma cells of the xylem and released into the xylem vessels (Hartung et al., 2002). Due to the low permeability of plant membranes, release into the xylem vessels represented a rate-limiting step (Baier et al., 1990). Owing to the very low permeability of plant membranes to ABA-GE, attributed to its highly hydrophilic nature, ABA-GE could undergo transport through the stem xylem without causing any loss to surrounding parenchyma tissues (Jiang and Hartung, 2008). Thus, ABA-GE could undergo long-distance transport under any conditions. During transport, ABA concentration did not remain constant. Under non-stress conditions, when the internal environment of the xylem parenchyma becomes acidic (due to the presence of abundant easily permeable undissociated ABAH), significant ABA loss may occur during long-distance transport within the xylem. Therefore, ABA-GE emerged as the most suitable molecule for providing hormone stress signals (Sauter and Hartung, 2002; Jiang and Hartung, 2008).

From the aforementioned facts, it can be concluded that the ABA transport system is highly complex and redundant. Plants have evolved intricate mechanisms to ensure correct and rapid responses to environmental stimuli under both normal and stress conditions. While ABA biosynthesis and signal transduction have been extensively and comprehensively studied, with researchers having gained a broad understanding of their molecular basis, the regulation of ABA transport networks has not been investigated in as much depth. Moreover, the species analyzed thus far were limited to *Arabidopsis*, rice, wheat, and similar plants. In the authors’ view, more attention should be paid to ABA transport. With the advent of advanced technologies such as cryo-electron microscopy for structural analysis of ABA transport proteins (Ying et al., 2023), recent studies have proposed a “gate-flipper” translocation model for ABA transmembrane transport mediated by ABCG25, providing insights into identification and transport mechanisms (Huang et al., 2023). These advancements will aid in better understanding the ABA transport network in plants.

## 7 Prospective

Reducing environmental stress is crucial for enhancing crop yields. A well-known function of ABA is its ability to improve plant tolerance to abiotic stress. Therefore, chemically regulating ABA signaling is an effective strategy in agricultural practice to mitigate the adverse effects of various stresses on crops. ABA has been widely registered for various applications in agriculture. Currently, it is approved for use in reducing transpiration; promoting fruit ripening, drought resistance, and hybrid seed production; and enhancing plant tolerance to transplantation, among other purposes (Nguyen et al., 2023). However, the biosynthesis of ABA is influenced by environmental factors, which limits its stability and effectiveness within plants. Through research on the ABA network, scientists have developed ABA receptor agonists. These compounds can mimic the natural functions of ABA and are used to study its biological roles or to enhance plant stress resistance. For example, the ABA mimic 1 (AM1), a potent activator of several members of the ABA receptor family, can simultaneously activate multiple ABA receptors, thereby protecting plants from water loss and drought stress (Cao et al., 2013). Incorporating a fluorine atom into the benzyl ring of AM1 can optimize its binding to ABA receptors by increasing the number of hydrogen bonds between the compound and the surrounding amino acid residues in the receptor’s ligand-binding pocket. This new compound, known as AMF, exhibits a prolonged effect in promoting stomatal closure and inducing the expression of stress-responsive genes (Cao et al., 2017). Using chemogenetics, scientists identified and optimized an ABA receptor agonist scaffold called opabactin. This compound is active in various monocots and eudicots, significantly enhancing drought resistance in plants (Vaidya et al., 2019). It is precisely due to the difficulties in synthesizing and purifying ABA, along with its rapid decomposition under natural light, that it has not been as widely used in agriculture as other plant hormones. Therefore, the search for ABA functional analogs that can enhance drought resistance in plants holds significant application potential and market value. In contrast to agonists, ABA receptor antagonists block the binding of ABA to its receptors, inhibiting ABA signal transduction and thereby regulating plant growth. Antabactin (ANT), an effective ABA blocker, disrupts signaling by stabilizing ABA receptors in a non-productive form, which can accelerate seed germination in various species, making it a valuable chemical tool for promoting germination (Vaidya et al., 2021). In conclusion, ABA receptor agonists and antagonists offer an efficient and sustainable approach to enhancing crop productivity and environmental adaptability in modern agricultural production. As these technologies continue to evolve and improve, they are expected to play an increasingly important role in global agriculture.

Furthermore, the application of ABA signaling pathways in crop improvement is gaining increasing attention, particularly in enhancing crop stress resistance. With a deeper understanding of ABA signaling, scientists have begun to leverage this pathway to develop crop varieties that are better equipped to withstand environmental stresses. For example, overexpressing ABA receptors in wheat has been shown to increase sensitivity to ABA, leading to reduced transpiration and enhanced photosynthesis in transgenic plants, thereby increasing yield per liter of water. Importantly, this improvement does not negatively affect plant growth and yield under normal conditions (Mega et al., 2019). This represents an ideal drought-resistant strategy for plants, which is crucial for maintaining high crop productivity in regions with limited water resources. There are also ABA-induced strategies for crop improvement, such as the *Deeper Rooting 1* (*DRO1*) gene in maize, which can be induced by ABA and plays a positive regulatory role in drought resistance. Using a synthetic ABA-inducible promoter to drive the expression of ZmDRO1 in modern maize, field grain yields under water-limited conditions have been successfully increased (Feng et al., 2022). With the advancement of molecular biology techniques, scientists can precisely manipulate key genes related to the ABA signaling pathway in crops through molecular marker-assisted selection, gene editing (such as the CRISPR-Cas system), and transgenic technologies. These approaches not only enhance crop stress resistance but also increase yield and improve quality by optimizing physiological and biochemical traits in crops. Two key genes involved in the ABA pathway were introduced into maize through *Agrobacterium*-mediated transformation. In a potted experiment under dehydration stress, non-transgenic control plants exhibited wilting and complete desiccation after prolonged stress, whereas the transgenic lines showed no signs of wilting (Muppala et al., 2021). This demonstrates that significant progress has been made in crop improvement through in-depth research and application of the ABA signaling pathway. In the future, as our understanding of ABA and its roles in plant physiology becomes more comprehensive, it is expected that more high-yielding, efficient, and stress-resistant crop varieties will be developed, contributing significantly to global food security and sustainable agricultural development.

In recent years, with the exploration of genomic and transcriptomic data in ABA synthesis species, the complete regulatory network of ABA biosynthesis, degradation, signal transduction, and transport regulation has gradually been elucidated. Some key genes involved in ABA regulation have also been identified through various overexpression or mutant studies. Despite the establishment of core ABA signaling pathways and models, breakthroughs have been made in ABA-related metabolism research, yet the ABA signal transduction network remained quite complex, and whether it could fully explain all ABA response mechanisms was unclear. Many questions remained to be explored: researchers discovered an ABA biosynthesis pathway independent of ZEP, raising the question of whether other synthetic pathways exist in plants to respond to environmental stress; 8′-hydroxylation is the main hydroxylation metabolic pathway, with its downstream products being extensively studied, but the downstream regulatory networks of the other two metabolic pathways have not been thoroughly analyzed. The relationship between transcription factors and downstream genes in plant ABA signal transduction still needs to be determined; questions regarding the regulation of ABA signal stability by sugars, amino acids, and other compounds also need to be addressed.

The application of high-throughput sequencing technology has greatly enhanced the exploration of ABA regulatory networks in various species. The development of cryo-electron microscopy has enabled scientists to understand the structure of proteins, proposing models for the roles of key proteins in the structural biology of ABA regulatory networks. The intersection of multi-omics and CRISPR-Cas gene editing technology, or more plant mutants, has laid the foundation for advancing research on the molecular mechanisms of ABA regulation. In the future, it may be possible to artificially regulate ABA signaling pathways through protein engineering and chemical molecule design. In conclusion, the application of ABA signaling pathways in agriculture and biotechnology has entered a new stage.
